# Direct bone marrow injection of human bone marrow-derived stromal cells into mouse femurs results in greater prostate cancer PC-3 cell proliferation, but not specifically proliferation within the injected femurs

**DOI:** 10.1186/s12885-022-09430-6

**Published:** 2022-05-17

**Authors:** Bianca Nowlan, Elizabeth D. Williams, Michael Robert Doran

**Affiliations:** 1 School of Biomedical Science, Faculty of Health, Queensland University of Technology at the Translational Research Institute, Brisbane, Australia; 2 Australian Prostate Cancer Research Centre-Queensland, Brisbane, Australia; 3 Centre for Biomedical Technologies, Queensland University of Technology, Brisbane, Australia; 4 Mater Research Institute - University of Queensland, Brisbane, Australia; 5 Skeletal Biology Section, National Institute of Dental and Craniofacial Research, National Institutes of Health, Bethesda, USA

**Keywords:** Prostate cancer, Bone marrow, Bone marrow mesenchymal stem cell, Bone marrow stromal cell, Mouse models, Humanization, Metastasize

## Abstract

**Background:**

While prostate cancer (PCa) cells most often metastasize to bone in men, species-specific differences between human and mouse bone marrow mean that this pattern is not faithfully replicated in mice. Herein we evaluated the impact of partially humanizing mouse bone marrow with human bone marrow-derived stromal cells (BMSC, also known as "mesenchymal stem cells") on human PCa cell behaviour.

**Methods:**

BMSC are key cellular constituents of marrow. We used intrafemoral injection to transplant 5 × 10^5^ luciferase (Luc) and green fluorescence protein (GFP) expressing human BMSC (hBMSC-Luc/GFP) into the right femur of non-obese diabetic (NOD)-severe combined immunodeficiency (*scid)* interleukin (IL)-2γ^−/−^ (NSG) mice. Two weeks later, 2.5 × 10^6^ PC-3 prostate cancer cells expressing DsRed (PC-3-DsRed) were delivered into the mice via intracardiac injection. PC-3-DsRed cells were tracked over time using an In Vivo Imaging System (IVIS) live animal imaging system, X-ray and IVIS imaging performed on harvested organs, and PC-3 cell numbers in femurs quantified using flow cytometry and histology.

**Results:**

Flow cytometry analysis revealed greater PC-3-DsRed cell numbers within femurs of the mice that received hBMSC-Luc/GFP. However, while there were overall greater PC-3-DsRed cell numbers in these animals, there were not more PC-3-DsRed in the femurs injected with hBMSC-Luc/GFP than in contralateral femurs. A similar proportion of mice in with or without hBMSC-Luc/GFP had bone lessions, but the absolute number of bone lesions was greater in mice that had received hBMSC-Luc/GFP.

**Conclusion:**

PC-3-DsRed cells preferentially populated bones in mice that had received hBMSC-Luc/GFP, although PC-3-DsRed cells not specifically localize in the bone marrow cavity where hBMSC-Luc/GFP had been transplanted. hBMSC-Luc/GFP appear to modify mouse biology in a manner that supports PC-3-DsRed tumor development, rather than specifically influencing PC-3-DsRed cell homing. This study provides useful insights into the role of humanizing murine bone marrow with hBMSC to study human PCa cell biology.

**Supplementary Information:**

The online version contains supplementary material available at 10.1186/s12885-022-09430-6.

## Background

Prostate cancer (PCa) is the second most common cancer in men [1]. While the 5-year survival rate for men with localized PCa is 99%, for patients with metastatic disease this decreases to 28% [1]. Of those who suffer metastatic disease, most (90.3%) will have bone metastasis [2]. When human PCa cells are transplanted into immune-compromised mice, metastasis to mouse bone does not occur with the same propensity as observed in humans [3, 4]. This disconnect is thought to reflect species-species differences between human and mouse bone marrow [5, 6]. The notion that the bone marrow is fundamentally different is supported by the observation that many human leukemias fail to engraft into mouse bone marrow, and that healthy human hematopoietic stem progenitor cells (HSPC) behave abnormally when engrafted into mouse marrow [7–9].

Bone marrow-derived stromal cells (BMSC, also known as “mesenchymal stem cells”) are viewed as a critical component of the bone marrow microenvironment [10]. BMSC are known to have a direct impact on HSPC engraftment and PCa cell metastasis [10–12]. Mouse and human BMSC have known species differences [13–15]. As BMSC play a critical role in the bone marrow microenvironment, BMSC species differences are likely to contribute to the different behaviour of PCa cells with respect to human and mouse marrow. In studies where ectopic bone marrows were established from human stromal cells, PCa cells populated the humanized marrows preferentially over mouse marrow [3, 6]. These data suggest that partially humanized marrow functions as a superior model for studying human disease, relative to native mouse marrow. In a variation on this theme, researchers have populated mouse marrow cavities with human stromal cells, and observed that human HSPC preferentially populated the humanized femurs [16–18]. For example, in a study reported by Carrancio *et al*., human BMSC (hBMSC) were directly transplanted into the femurs of NOD/SCID mice, and human HSPC transplanted either by co-injection into the femurs or via intravenously [19]. Greater human HSPC engraftment was observed in femurs populated by hBMSC. hBMSC were found only in the femurs that they had been directly injected into, suggesting that this was a viable method for establishing hBMSC population localized within a mouse bone marrow cavity. We reasoned that a similar model of direct injection of hBMSC into the marrow cavities of mice could be used to facilitate the study of human PCa cells.

Herein we partially humanized mouse bone marrow cavities, as previously described [20], by injecting 5 × 10^5^ luciferase (Luc) and green fluorescence protein (GFP) expressing hBMSC (hBMSC-Luc/GFP) into the right femur of NOD/*scid* IL2γ^−/−^ (NSG) mice. After allowing animals to recover for 2 weeks, 2.5 × 10^6^ DsRed labelled PC-3 human PCa (PC-3-DsRed) cells were delivered into mice via intracardiac injection. We tracked hBMSC-Luc/GFP and PC-3-DsRed location and number in live animals with an In Vivo Imaging System (IVIS) system for 4 weeks. Animals were sacrificed, and PC-3-DsRed tumor formation was characterized by X-ray, harvested organs characterized using IVIS, and cell number in femurs estimated using flow cytometry and histology.

## Methods

### hBMSC-Luc/GFP cells

The collection and use of human bone marrow was approved by the Mater Hospital Human Research Ethics Committee and by the Queensland University of Technology Human Research Ethics Committee (Ethics No.: 1000000938). Volunteer donors provided informed written consent, and all processes followed the National Health and Medical Research Council of Australia guidelines. hBMSC from two donors were used to optimize direct bone marrow injection. Finally, hBMSC from a 22-year-old male donor were used in the PCa cell studies described here. hBMSC were isolated and cultured as previously described by our team [21]. Unless specified, all cell culture reagents were sourced from Thermo Fisher Scientific (Massachusetts, USA). hBMSC were enriched for by plastic adherence and expanded in medium formulated from low glucose Dulbecco’s Modified Eagle’s Medium (LG-DMEM), 10% fetal bovine serum (FBS), 1% penicillin/streptomycin (P/S) and 10 ng/mL fibroblast growth factor-1 (FGF-1, Peprotech, Rehovot, Israel). Cultures were maintained in a humidified 2% O_2_ and 5% CO_2_ incubator.

hBMSC were transduced to express GFP and luciferase (hBMSC-Luc/GFP) as previously described [20]. In brief, a third-generation lentiviral system was used to integrate the Luc/GFP genes, where expression was driven by a Murine Stem Cell Virus promotor (MSCV, System Bioscience, pBLIV301PA-1, California, USA). Viral particles were produced using HEK293T cells, with the Luc/GFP construct delivered in combination with the TGEN packaging plasmid mix at a ratio of 1:3 (μg DNA: μL reagent) in Lipofectamine 2000 (Thermo Fisher Scientific). Medium containing viral particles was collected and used to transduce hBMSC. Three days later, GFP^+^ hBMSC-Luc/GFP were enriched for by flow cytometry sorting (Beckman Coulter Astrios, California, USA), and these cells further expanded in culture. Experiments were performed using passage 4–6 hBMSC-Luc/GFP.

### PC-3-DsRed *cells*

PC-3 expressing pDsRed2-N1 cells (PC-3-DsRed, Supplementary Fig. 1) were transduced as described previously [22]. In brief, parental PC-3 cells were transduced with pDsRed2-N1 (BD Biosciences, cat no. 632406, New Jersey, USA). PC-3-DsRed were cultured in high glucose DMEM (HG-DMEM, Gibco) supplemented with 10% FBS and 1% P/S. Cells were tested for stability without selective vector pressure by culturing with or without 800 μg/mL G418 (Merck). Cells were characterized on a Beckman Coulter Cytoflex to measure the relative fluorescent signal from PC-3-DsRed, with or without selection pressure, and from a control (non-transduced) PC-3 cell population. Analysis of data was performed with FlowJo v10 software (BD Biosciences). Cell fluorescence was validated using microscopy, and titrations of cells in a 96 well plate used to demonstrate that a linear signal, relative to cell number, could be acquired with an IVIS.

### Animal handling and ethics

All animal work was designed and approved as per the National Health and Medical Research Council of Australia guidelines. Animal breeding and procedures were approved by the University of Queensland Animal Ethics Committee and by the Queensland University of Technology (QUT) Ethics Committee. NOD-scid IL2γ^−/−^ (NSG) mice breeding pairs were purchased from Jackson Laboratories (Stock No. 001976, Maine, USA), and animals bred at the Translational Research Institute Biological Research Facility (Brisbane, Australia). Mice were maintained on ad-lib standard chow and water in standard conditions with a 12-h light/dark cycle. Male mice, 6–8 weeks old, were used in these studies. Mice were average weight of 28.3 g (22.1–34.5 g) at the start of experiment.

### Transplant of hBMSC-Luc/GFP and injection of PC-3-DsRed

Mice were conditioned with 2 Gy γ-total body irradiation (137Cs, Gammacell 40 Exactor, Best Theratronics). On the following day, mice were allocated to groups and administrated anesthesia of Ketamine (75 mg/Kg) and Xylazine (15 mg/Kg). hBMSC-Luc/GFP (5 × 10^5^) were resuspended in X-VIVO 10 (Lonza, Basel, Switzerland). Cells were injected into the right femur of mice using a previously described protocol [23]. Mice were given analgesia (Buprenorphine, 0.03 mg/kg) the day of injection and the next day. Two weeks after hBMSC-Luc/GFP transplant, saline or 2.5 × 10^6^ PC-3-DsRed were delivered via intracardiac injection. Mice were assigned a group using a random number generator to assign injection order. Four animal groups were established: (1) no cells, (2) PC-3-DsRed only, (3) hBMSC-Luc/GFP only, and (4) hBMSC-Luc/GFP + PC-3-DsRed as outlined in Supplementary Fig. 2. Intracardiac injection was performed with animals anesthetised with isoflurane. Mice were monitored for health and weight.

### IVIS imaging of animals

Animals were imaged immediately following injection of hBMSC-Luc/GFP, and at weekly intervals afterwards. Bioluminescence was used to detect hBMSC-Luc/GFP, and fluorescence signal used to detect PC-3-DsRed.

Bioluminescence signal was acquired while the animals were sedated following hBMSC-Luc/GFP and D-luciferin injection (imaging 10 min post-D-luciferin injection, 150 mg/Kg, Perkin Elmer, New Jersey, USA). Bioluminescence data required a region of interest (ROI) to be drawn around the injected femur. In some mice (9/19, 47.4%) we observed a bioluminescence signal in the lungs immediately following transplant. These animals were initially analyzed separately (Supplementary Fig. 3) to determine if this influenced results, and subsequently all data sets were combined in the final analysis.

DsRed fluorescence signal was captured used the IVIS dual filter method (excitation background 500 nm or DsRed 570 nm, emission filter 620 nm, Supplementary Fig. 4) at injection and each week following. Mice that displayed an elevated DsRed signal in the heart at week zero were excluded from further analysis. The relative DsRed fluorescent signal was estimated using the Live Image Math algorithms (Perkin Elmer), subtracting the background signal from a no cell control animal with each image. To quantify the fluorescence signal, we utilized the auto-threshold determination of ROI set at 15% to non-bias detection of fluorescence (Supplementary Fig. 4). Where multiple ROIs were measured per mouse, these values were combined during analysis.

### Tissue harvest

Mice were euthanized (carbon dioxide), and imaged using X-ray (Faxitron, Hologic, Arizona, USA). Legs, liver, lung, and spleens were harvested, laid out in petri-dishes, and PC-3-DsRed signal captured with the IVIS. Tissue cell content was subsequently further characterized by flow cytometry, or tissues fixed in 4% paraformaldehyde (PFA, Sigma-Aldrich) overnight for histological analysis.

### Histology

All antibodies used in this project are listed in Supplementary Table 1. Bones were decalcified with 15% ethylenediaminetetraacetic acid (EDTA, Merck) plus 0.5% paraformaldehyde in phosphate-buffered saline (PBS). Decalcified tissues were then dehydrated in ethanol (16 h) and embedded in paraffin. Paraffin sections (5 μm) adhered to a Super Frost slide, and slides were set at 50 °C for 1 h to assist in adhesion. Slides were de-paraffined with exchanges of xylene, and then rehydrated in dilutions of ethanol into PBS. Tissue slices were stained with hematoxylin and eosin (H&E) or with antibodies.

In preparation for antibody staining, antibody retrieval was performed by treating tissue slices in citrate buffer (10 mM Sodium Citrate, 0.05% Tween 20, pH 6.0, Merck) for 20 min in a 95 °C water bath. Samples were then blocked with Background Sniper (Biocare Medical, Cat no. BS966, California, USA) reagent according to manufacturer instructions and stained overnight with chicken anti-GFP or primary antibody omitted as a control. Samples were then washed with Tris-buffered saline with 0.05% Tween-20 and stained with donkey anti-chicken Alexa Fluor 647. Samples were then washed and stained for 10 min with 1 μg/mL 4′, 6-diamidino-2-phenylindole (DAPI, Thermo Fisher Scientific, Cat no. D1306) for nuclei identification, and coverslipped using Prolong Gold (Thermo Fisher Scientific, Cat no. P36934).

Slides were imaged on a 3DHISTECH Slide Scanner (Budapest, Hungary) at 20X magnification. Resultant images were analyzed on the Case Viewer (V2.2, 3DHISTECH) and staining quantified using ImageJ [24]. Slides were imaged using autofocus and the auto acquisition protocol. Background fluorescence was quantified by scanning an unused channel, and these data were used to threshold the sample. The number of hBMSC-Luc/GFP was estimated by acquiring three random images of the bone marrow and counting the number of events that were GFP^+^ and DAPI^+^, relative to the total DAPI^+^ events.

### Flow cytometry analysis

Injected and contralateral femurs were analyzed separately. Femurs were gently crushed, and treated with 3 mg/mL Collagenase Type I (Worthington, New Jersey, USA) for 40 min at 37 °C. Cells were separated from debris by passing through a 40 μm strainer. Cells were stained with anti-mouse CD45 and the live-dead discriminator 7-amino-actinomycin D ((7-AAD) Merck, 20 μg/mL, Cat no. A1310), and analyzed on a Beckman Coulter Cytoflex to detect and quantify the relative number of PC-3-DsRed. Analysis of data was performed with FlowJo v10 software.

### Statistics

Mice were masked with the mouse number during image selection and processing. Mice groups were only unmasked after analysis. All statistics were completed using GraphPad Prism 8 (La Jolla, CA) after column statistics were used to select the correct test. The ROUT test was used to identify outliers in analysis. Reported numbers are group average ± one standard deviation. Linear regression was used on repeated measurements to determine group differences with fit-test completed using Alkaines Information Criterion (AICc). Paired comparisons were completed with Mann-Whitney t-tests.

## Results

### hBMSC-Luc/GFP and PC-3-DsRed imaging in live animals

**Fig. 1:**
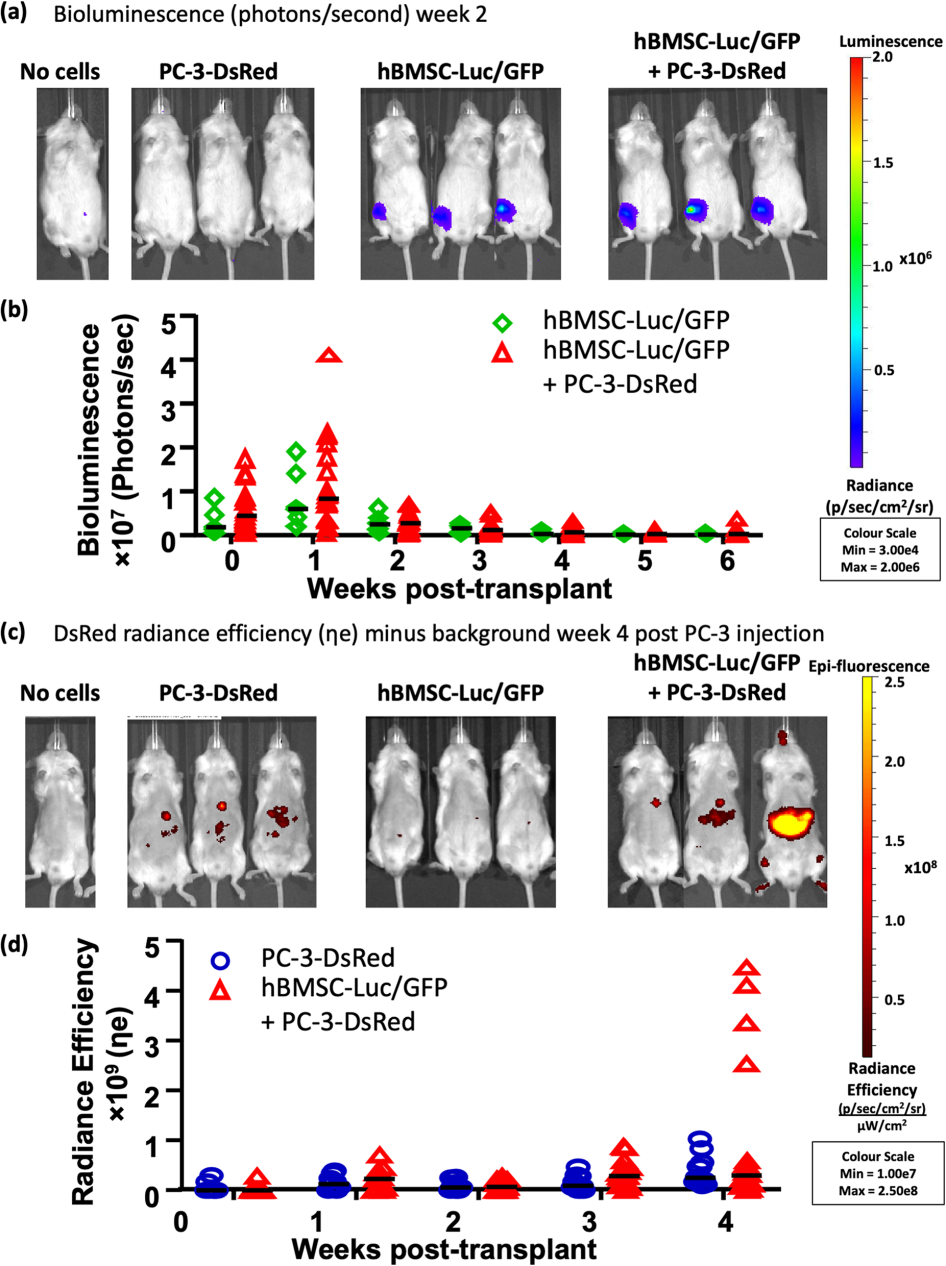
Live animal IVIS imaging. (**a**) Bioluminescence signal from representative mice that received hBMSC-Luc/GFP (image time point was two weeks after transplant). (**b**) Graphical representation of bioluminescence hBMSC-Luc/GFP signal overtime for animals that did or did not receive PC-3-DsRed injections (8 mice with hBMSC-Luc/GFP (green), and 18 mice with hBMSC-Luc/GFP + PC-3-DsRed (red)). (**c**) Fluorescence signal from PC-3-DsRed, minus background fluorescence, for select mice from each group at 4 weeks (14 mice with PC-3-DsRed and 18 mice with hBMSC-Luc/GFP + PC-3-DsRed). (**d**) Graphical representation of PC-3-DsRed fluorescence signal from mice overtime after PC-3-DsRed injection. Pooled experiments of three biological repeats. All IVIS images are found in Supplementary Figs. 5 and 7. Statistics were not significant between curves after using linear-regression calculation and fit determined by Alkaines Information Criterion (AICc) or multiple t-tests with the Holm-Sidak method (Supplementary Fig. 6).

Mice were injected with media or hBMSC-Luc/GFP 24 h after 2 Gy total body irradiation. hBMSC-Luc/GFP signal from the injected femurs tapered with time but remained visible at 6 weeks post-transplant (Fig. 1a-b, Supplementary Fig. 5). At the time of hBMSC-Luc/GFP transplant, a bioluminescence signal could be detected in the lungs of some animals, however, by the time of PC-3-DsRed injection; bioluminescence signal could only be detected as emanating from the injected femurs. Previous studies demonstrate that hBMSC entrapped in the lungs of mice are rapidly cleared [25], and this is consistent with our IVIS imaging. The analysis was completed with and without animals that had a transient bioluminescence signal from the lungs (Supplementary Fig. 3), and based on the similarity of results, data from all animals was pooled for the primary analysis in this paper. PC-3-DsRed cells were injected into mice at 2 weeks post-hBMSC-Luc/GFP transplant.

Analysis of IVIS images indicated no difference in hBMSC-Luc/GFP bioluminescence signal between animals that received PC-3-DsRed or those that did not (Fig. 1b and Supplementary Fig. 6a). In Supplementary Fig. 6a, AICc fit-test was used to estimate the probability that a single curve fit bioluminescence data from mice with or without PC-3-DsRed. This analysis suggested that the presence of PC-3-DsRed cells did not influence the growth of hBMSC-Luc/GFP in mice.

PC-3-DsRed fluorescence signal was also monitored with IVIS (Fig. 1c-d, Supplementary Fig. 7). Signal was variable between animals, likely due to the exponential expansion of PC-3-DsRed in some animals, although greater signal was derived from animals that had received hBMSC-Luc/GFP. AICc fit-test was used to estimate the probability that a single curve fit PC-3-DsRed fluorescence signal data from animals with or without hBMSC-Luc/GFP, and this was found to be unlikely suggesting that the presence of hBMSC-Luc/GFP did influence PC-3-DsRed numbers (Linear regression, AiCc = 55.06%, Supplementary Fig. 6b).

### Spatial quantification of hBMSC-Luc/GFP and PC-3-DsRed

**Fig. 2:**
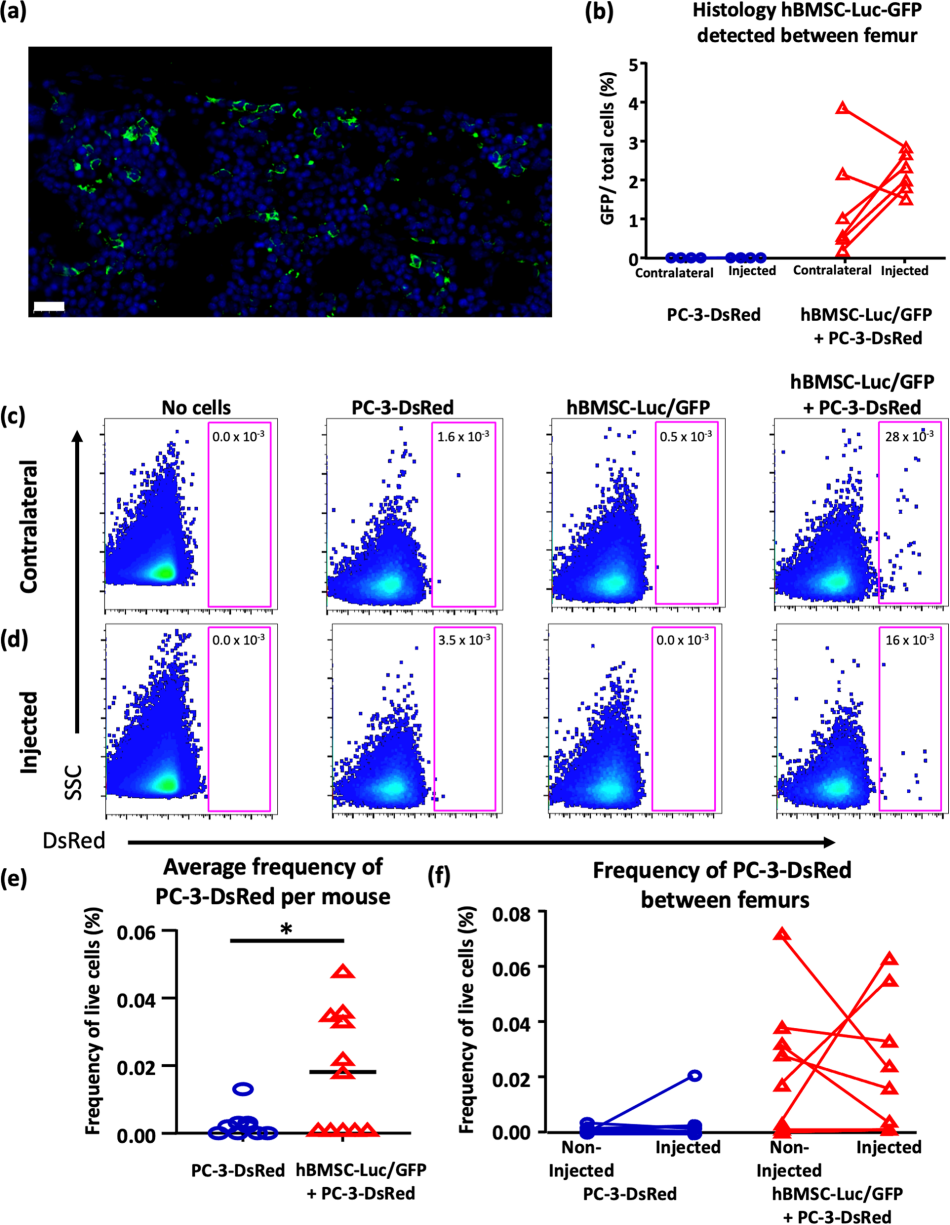
Analysis of hBMSC and PC-3 by histology and flow cytometry. **(a, b)** Quantification of hBMSC-Luc/GFP in femur histology slices. **(a)** Histology 40x magnification image of marrow with anti-GFP (green) and DAPI (blue) to detect hBMSC-Luc/GFP. Scale bar = 20 μm. **(b)** Comparison of relative hBMSC-Luc/GFP numbers in histology slices at 6 weeks (PC-3-DsRed *n* = 4, hBMSC-Luc/GFP + PC-3-DsRed *n* = 6). Flow cytometry quantification of PC-3-DsRed numbers in **(c)** mouse contralateral and **(d)** injected femurs. Gating identified live singlet cells, which were negative for mouse CD45, but positive for a DsRed signal (Supplementary Fig. 9). **(e)** Quantification of total PC-3-DsRed numbers taking the average of both femurs, in animals that either did or did not receive hBMSC-Luc/GFP. Statistics determined by the Mann-Whitney t-test detected a significant difference (*p* = 0.0445) in the number of PC-3-DsRed in animals that had been transplanted with hBMSC-LUC/GFP. **(f)** Comparison of the distribution of PC-3-DsRed between femurs in individual mice femurs. Individual flow images are found in Supplementary Fig. 10. Mann Whitney t-test did not identify difference between injected vs non-injected femur (PC-3-DsRed, *p* = 0.6589; hBMSC-Luc/GFP + PC-3-DsRed, *p* = 0.5223). Two flow experiments pooled, (no cells *n* = 2, PC-3-DsRed only *n* = 8, hBMSC-Luc/GFP only *n* = 7, hBMSC-Luc/GFP + PC-3-DsRed *n* = 11).

We used histology to identify and quantify hBMSC-Luc/GFP within the femurs of mice at harvest. As previously reported [20], we detected the GFP^+^ cells in both in the injected femurs and in the contralateral femurs, indicating that hBMSC-Luc/GFP had disseminated to other marrow cavities (Fig. 2a, b). Previous studies reported that intravenously transplanted hBMSC home and engraft within the bone the marrow of mice [26, 27]. Immediately following hBMSC-Luc/GFP transplant, a bioluminescence signal emanating from the lungs could be seen in some mice, demonstrating that detectable numbers of cells had escaped from the bone marrow cavity into the general circulation, and we presume that some of these cells homed to distal bone marrow cavities. In histological sections of injected and contralateral femurs, 6 week after initial transplant, the difference between the hBMSC-Luc/GFP numbers in these marrow cavities was insignificant (injected femur 2.2 ± 0.5% versus contralateral femur 1.4 ± 1.4%, Mann-Whitney t-test, *p* = 0.1797). We did not detect a change in cellularity of femurs that were injected with hBMSC-Luc/GFP compared to either the contralateral femur or femurs from mice that did not receive hBMSC at all (student t-test, *p* = 0.5898). This indicated that the hBMSC transplant did not cause a detectable long-term impact on marrow cellularity (Supplementary Fig. 8).

The number of PC-3-DsRed in each femur was quantified using flow cytometry. PC-3-DsRed were identified as viable cells (7-AAD^−^), negative for mouse CD45, and positive for DsRed (see Gating strategy in Supplementary Fig. 8). PC-3-DsRed were detected (higher than 0.01% of live CD45^−^ cells) in 1 out of 10 mice in the PC-3-DsRed only group (12.5%), compared to 6 out of 11 in the hBMSC-Luc/GFP + PC-3-DsRed group (54.5%). The hBMSC-Luc/GFP + PC-3-DsRed group had an additional mouse that had 5-fold greater PC-3-DsRed burden. This animal was considered an outlier and excluded from subsequent analysis. hBMSC-Luc/GFP + PC-3-DsRed mice had an overall higher PC-3-DsRed burden in femurs (Fig. 2e, 0.018 ± 0.018% vs 0.002 ± 0.003%, Mann-Whitney t-test with a 95% confidence *p* = 0.0445, individual flow plots Supplementary Fig. 10). There was not a greater frequency of PC3-DsRed in the specific humanized femur relative to the contralateral femur in the same animal that had not been injected with hBMSC-Luc/GFP (Fig. 2f, Mann-Whitney t-test, *p* = 0.5223). In summary, the presence of hBMSC-Luc/GFP in the animal increased the frequency of PC-3-DsRed detected in the femurs, but PC-3-DsRed cells did not specifically localize in the femur where hBMSC-Luc/GFP had been initially transplanted.

### PC-3-DsRed tumor burden in the bone marrow and visceral tissue

**Fig. 3:**
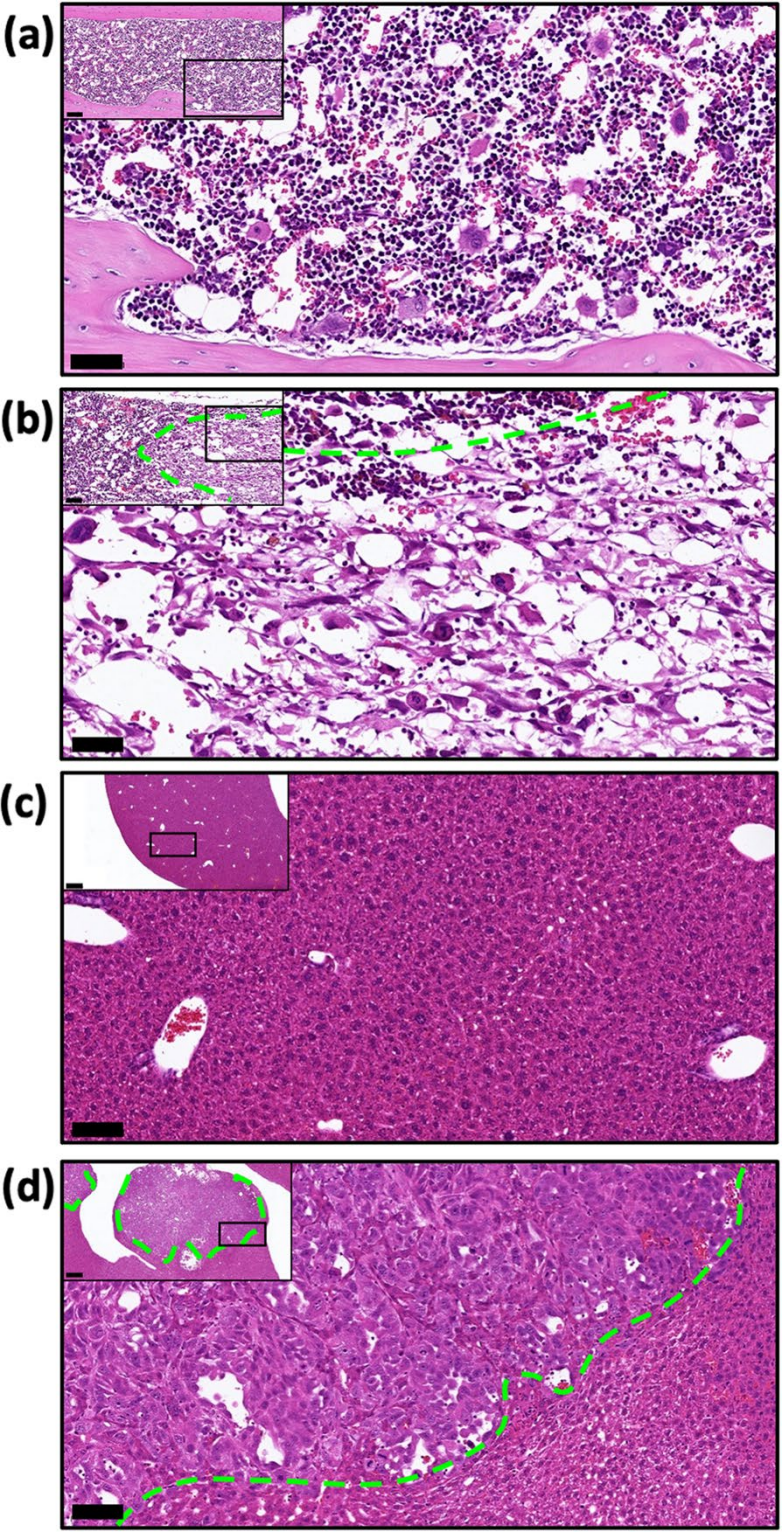
H&E stained histology sections of bone marrow and liver from healthy animals’ versus those injected with PC-3-DsRed. **(a)** Normal bone marrow with regular cellular morphology (scale bar 50 μm), and a lower magnification inset (scale bar 100 μm), in comparison to (**b**) bone metastasis with irregular cellularity from the hBMSC + PC3 group. (**c**, **d**) Liver section from normal verses liver metastasis (scale bar 100 μm, inset 500 μm). Green dashed lines represent the border between tumor tissue and normal tissue.

Tissue sections were stained with H&E. Regions containing PC-3-DsRed cells were selected for analysis in samples from mice injected with tumour cells. Characteristic irregular cell morphology was visible in the bone marrow (Fig. 3a, b, normal versus tumor-bearing) and in the liver (Fig. 3c, d, normal versus tumor-bearing).

**Fig. 4:**
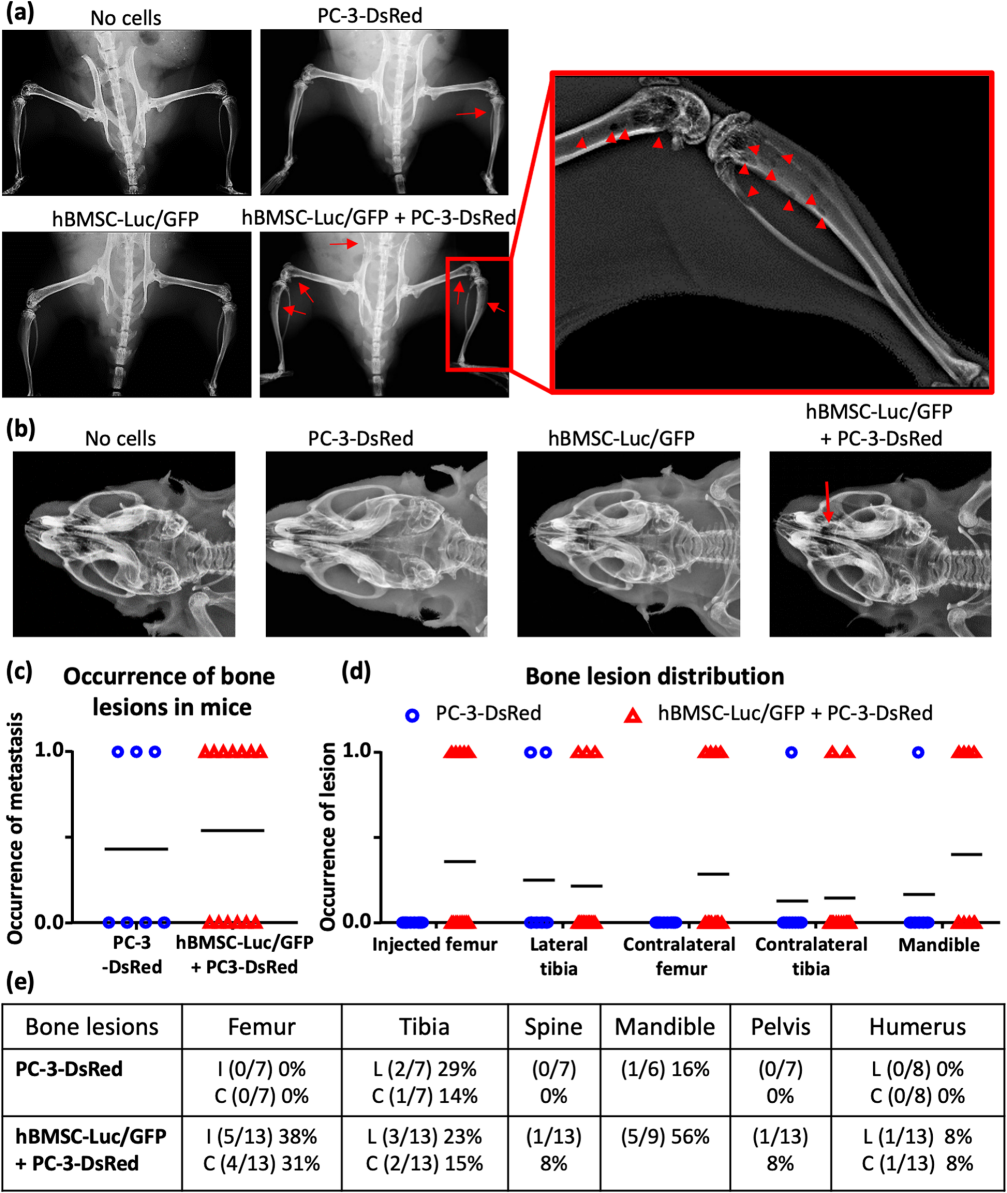
hBMSC-Luc/GFP influence on the frequency of PC-3-DsRed bone lesions. (**a**) X-ray of mouse legs, or (**b**) skull of a representative mouse from each group. Red arrows identify lesions. Mice were scored for lesion development with 1, or none with 0. (**c**) Graphed binary results of total bone lesions occurrence, versus (**d**) graphed lesions per location. (**e**) Table of occurrence and frequency of lesions detected at each bone site. All images are found in Supplementary Fig. 11 and 12. Acronyms: I: injected, C: contralateral, L: lateral; PC-3-DsRed *n* = 7, hBMSC-Luc/GFP + PC-3-DsRed *n* = 13.

X-ray imaging of mice (Fig. 4a-b, Supplementary Figs. 11 and 12) identified bone lesions in 4 out of 8 (43%) mice that received PC-3-DsRed, which was similar to mice that had received hBMSC-Luc/GFP + PC-3-DsRed (7/14, 54%, Binary analysis, Mann-Whitney t-test *p*= > 0.9999). Mice in the hBMSC-Luc/GFP + PC-3-DsRed group had a greater number of bones impacted (Fig. 4c-d, Mann-Whitney t-test *p* = 0.0238, Supplementary Table S2). For instance, hBMSC-Luc/GFP + PC-3-DsRed resulted bone lesions in the femurs, tibias, and mandible, and in one animal lesions were observed in the spine, pelvis, and humerus, while in the PC-3-DsRed only group lesions were only detected in the tibias and mandibles.

**Fig. 5:**
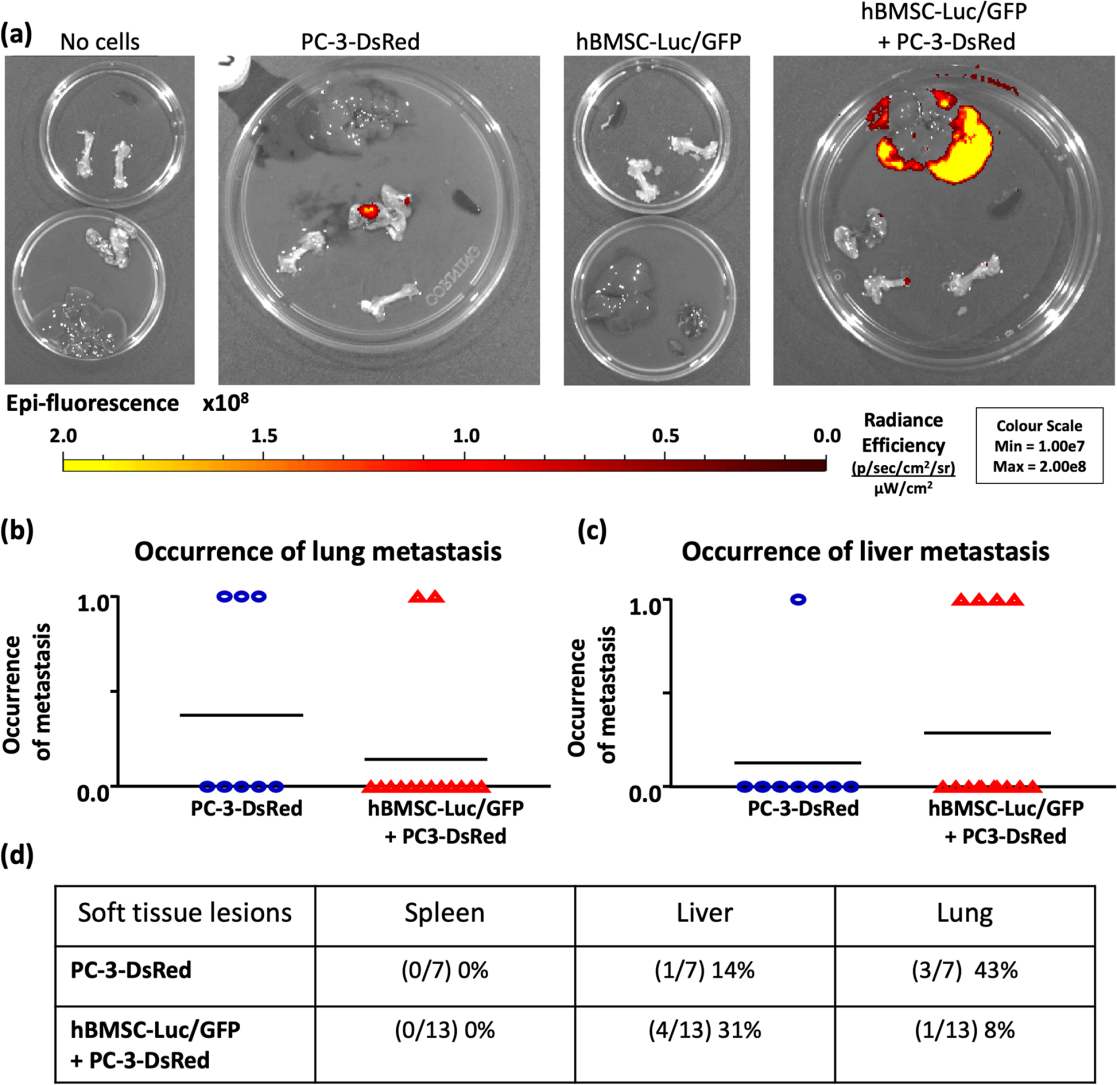
The frequency of PC-3-DsRed lesions. (**a**) IVIS image of DsRed (minus background) of representative mouse organs from each group. Mice were scored for lesions with 1, or none with 0, to generate the resultant graph of binary occurrence in the (**b**) lung or (**c**) liver. (**d**) Table of identified organ lesions and frequencies. Individual images are in Supplementary Fig. 13. PC-3-DsRed *n* = 7, hBMSC-Luc/GFP + PC-3-DsRed *n* = 13.

Harvested organs were imaged with an IVIS to detect lesions in these tissues (Fig. 5, Supplementary Fig. 13, and Supplementary Table 2). The PC-3-DsRed only group was found to have a non-significant trend of greater lung lesions than in the hBMSC-Luc/GFP + PC-3-DsRed group (3/7 43% vs 1/13 8%, Mann-Whitney t-test *p* = 0.1011). By contrast, the hBMSC-Luc/GFP + PC-3-DsRed group non-significantly trended towards a higher frequency of liver lesions (PC-3-DsRed 1/7 (14%) versus hBMSC-Luc/GFP + PC-3-DsRed 4/13 (31%), Mann-Whitney t-test *p* = 0.6126). Femurs were also imaged but rarely generated a DsRed signal, although bone lesions were detected as described earlier. Failure to detect a DsRed signal from bones likely reflects IVIS imaging limitations, which require a significant cluster of cells to generate a detectable signal, and the opaque nature of the bone tissue.

## Discussion

Species-species differences between mouse and human bone marrow limit the utility of mouse marrow as a model system to study human PCa [5, 6]. Herein we assessed if the direct injection of hBMSC into an established marrow cavity in mice would yield a chimeric mouse-human bone marrow that could be used to study human PCa cell behaviour. We transplanted hBMSC-Luc/GFP into the right femur of NSG mice. Two weeks later, PC-3-DsRed PCa cells were delivered via intracardiac injection.

Live animal imaging was used to track the bioluminescence signal from the hBMSC-Luc/GFP and the fluorescent signal from the PC-3-DsRed over the 6-week study. At the time of PC-3-DsRed injection, the bioluminescence signal from the hBMSC-Luc/GFP identified these cells as being concentrated within the injected femurs. The hBMSC-Luc/GFP bioluminescence signal from the injected femurs declined over time and was undetectable in some animals at 6 weeks. The presence or absence of PC-3-DsRed did not influence the hBMSC-Luc/GFP bioluminescence signal. By contrast, the live animal fluorescent signal from the PC-3-DsRed increased with time as the PCa cells increased in number. The PC-3-DsRed fluorescent signal was greater from animals that had been transplanted with hBMSC-Luc/GFP.

We used histology to quantify hBMSC-Luc/GFP numbers in the injected and contralateral femurs. While hBMSC-Luc/GFP numbers declined within injected femurs over the course of the 6-week study, at the endpoint a similar number of hBMSC-Luc/GFP were identified in the histological sections of both injected and contralateral femurs as previously reported by our team [20]. Previous studies reported that intravenously transplanted hBMSC home to murine bone marrow [26, 27], and a recent study suggests that a direct bone marrow injection volume of greater than 3 μL results in leakage from the NSG mouse femur cavity into the general circulation [28]. Nevertheless injection volumes of 10–40 μL are commonly used in direct bone marrow injection studies [23, 29–34]. The hBMSC cell suspension volume injected into femurs in our study was 10 μL. While the injected marrow functions as a sieve, retaining many of the injected cells, many cells may also escape into the general circulation. The prevalence of hBMSC in the distal marrow cavities of mice in our study is an indication of either loss into the general circulation during transplant, or that the hBMSC actively migrated from the injected marrow to distal marrow cavities. Cumulatively, these data suggest: (1) that a high local density of hBMSC-Luc/GFP within injected femurs can be achieved, but that this localized population declines with time, and (2) a portion of the injected hBMSC-Luc/GFP escape during transplantation and home to distal marrow cavities.

Using flow cytometry we quantified the number of PC-3-DsRed in both femurs of animals. The number of PC-3-DsRed was similar between femurs injected with hBMSC-Luc/GFP and distal femurs. However, a greater number of PC-3-DsRed were detected in both femurs of the mice that had received hBMSC-Luc/GFP transplants, relative to mice that had not received hBMSC-Luc/GFP transplants. There was also a trend towards a greater incidence of liver lesions in hBMSC-Luc/GFP transplanted mice and fewer lung lesions than in PC-3-DsRed only mice (non-significant). Although there was no change in the percentage of mice found to have bone lesions, the group that received hBMSC-Luc/GFP had a higher frequency of bone lesions than the group that did not receive hBMSC-Luc/GFP.

Analysis of the PC-3-DsRed data suggests that the presence of hBMSC-Luc/GFP more likely influenced lesion development, rather than causing PC-3-DsRed cells to home to a specific tissue. This observation aligns with previous reports indicating that many PCa cell populations rapidly migrate to mouse endocortical bone within 24 h after intravenous injection [35], but that only some PCa cell populations are capable of progressing into localized growths [5, 36–38]. Our data suggest that the general presence of hBMSC-Luc/GFP in the animal, rather than their specific location, facilitates PC-3-DsRed progression to form localized tumour growths. hBMSC express several soluble cytokines, chemokines, pro-angiogenic, anti-apoptotic, and anti-inflammatory signals that have been previously associated with PCa tumor progression [39–41], and the secretion of these molecules into the circulation of animals may generally encourage PCa cell growth. Previous regenerative medicine studies indicate that hBMSC exert paracrine effects on tissues distal from the physical location of transplanted cells [25, 42], and this pattern appears to be replicated in our study.

Previous studies observed that PC-3 cells form growths more frequently in the lungs (25–71.4%) than the liver (12.5–28.7%) [5, 43]. We observed the opposite pattern, with greater PC-3-DsRed growths in the liver (4/14, 28.6%) than in the lungs (2/14, 14.3%) of animals transplanted with hBMSC-Luc/GFP. Intravenously transplanted BMSC frequently become lodged in the lungs or liver of mice immediately following injection [44, 45], and we observed bioluminescence signal from the lungs of some animals, but did not detect hBMSC-Luc/GFP in the histology of the lung tissue at week 6. This aligns with studies that have reported transplanted BMSC become lodged in the lungs, but suggests that these BMSC are then rapidly cleared from the animal [25].

A primary goal of this study was to populate a mouse femur with hBMSC-Luc/GFP and use the contralateral femur as a control to study PC-3-DsRed homing. While IVIS bioluminescence imaging revealed a significant physical bias of hBMSC-Luc/GFP localization within the injected femur at the time of PC-3-DsRed injection, histological data suggested that relative hBMSC-Luc/GFP numbers were similar in both femurs at week 6. It is reasonable to assume that efforts to detect a bias in PC-3-DsRed numbers between femurs was obfuscated by the declining number of hBMSC-Luc/GFP within the injected femur, and the prevalence of hBMSC-Luc/GFP in contralateral femurs. The failure to maintain a significant number of hBMSC-Luc/GFP in the injected femur exposes a potentially major limitation with this model and approach to humanizing a mouse bone marrow cavity. Additionally, the dissemination of hBMSC-Luc/GFP to other marrow cavities compromises efforts to decouple assessment of the influence of hBMSC on human PCa cell homing and proliferation. Over the brief (4-week) periods we observed considerable variably in size and distribution of PC-3-DsRed lesions. It is likely that if this assay were extended, to allow tumors to grow, the variability would increase, and greater animal numbers would be required to detect differences. Despite model limitations, these data provide compelling evidence that hBMSC do alter PC-3 behaviour in NSG mice. Thus, while the assay did not perform as expected, these data justify further investment into efforts to humanize mice with hBMSC.

## Conclusion

Herein we established a new mouse model where a bone marrow cavity was partially humanized by transplanting hBMSC directly into the femur cavity. When PC-3 PCa cells were injected into mice, a greater number of these cells were found to be populating the bones of animals that had been transplanted with hBMSC. However, the number of PC-3 PCa cells was found to be similar in both the femur previously transplanted with hBMSC and the contralateral femur. These data suggest that the mechanism by which hBMSC promotes the formation of PC-3 lesions is via a paracrine secretions that generally upregulate the human cancer cell growth, rather than by influencing human cancer cell homing towards the transplanted hBMSC populations. Cumulatively, these data suggest that hBMSC do modify PC-3 behaviour in mice and that it may be possible to exploit hBMSC to generate superior animal models to aid PCa research.

## Supplementary Information

**Additional file 1:** **Supplementary Tables.**

## Data Availability

The datasets used and analyzed during the current study are available from the corresponding author on reasonable request.

## Abbreviations

7-AAD: amino-actinomycin D
AICc: Alkaines Information Criterion
APC: Allophycocyanin
A647: Alexa Fluor 647
Cy7: Cyanine dye − 7
BMSC: Bone marrow-derived stromal cells
DAPI: 6-diamidino-2-phenylindole
EDTA: Ethylenediaminetetraacetic acid
FBS: Fetal bovine serum
FGF-1: Fibroblast growth factor-1
GFP: Green fluorescence protein
H&E: Hematoxylin and eosin
hBMSC-Luc/GFP: green fluorescence protein (GFP) expressing human BMSC
HG-DMEM: High glucose DMEM
HSPC: Hematopoietic stem progenitor cells
IVIS: In Vivo Imaging System
Luc: Luciferase
MSCV: Murine Stem Cell Virus promotor
NOD: Non-obese diabetic
NSG mice: Non-obese diabetic (NOD)-severe combined immunodeficiency (*scid*) interleukin (IL)-2γ−/− mice
PC-3-DsRed: PC-3 prostate cancer cells expressing DsRed
PCa: Prostate cancer
PE: R-phycoerythrin
PFA: Paraformaldehyde
QUT: Queensland University of Technology
ROI: Region of Interest
*scid*: Severe combined immunodeficiency
TRI: Translational Research Institute
