# Supplementary material for: Direct bone marrow injection of human bone marrow-derived stromal cells into mouse femurs results in greater prostate cancer PC-3 cell proliferation, but not specifically proliferation within the injected femurs
Source: BMC Cancer. 2022 May 17;22:554. doi: 10.1186/s12885-022-09430-6 (PMC9112579; doi:10.1186/s12885-022-09430-6)
Supplement: Supplementary file 1 — Additional file 1: Supplementary Tables. [file 12885_2022_9430_MOESM1_ESM.docx]

**Supplementary Data**

**Supplementary Tables**

Supplementary Table 1. Antibodies used in experiments.

| Antibody | Catalogue number | Company | Concentration used |
| --- | --- | --- | --- |
| **Flow cytometry antibodies** |  |  |  |
| mCD45 APC | 559864 | Beckman Dickson | 1 µg/mL |
| h/mCD11b PE | 101208 | Biolegend | 1 µg/mL |
| mGr-1 (Ly6G/C) APC-Cy7 | 557661 | Beckman Dickson | 1 µg/mL |
| Fc Block | 553142 | Beckman Dickson | 0.1 µg/mL |
| **Histology antibodies** |  |  |  |
| Chicken anti-GFP | Ab13970 | Abcam | 40 µg/mL |
| Donkey anti-Chicken A647 | 703-065-155 | Jackson Immunology | 3 µg/mL |

Acronyms: h, Human; m, mouse; APC, Allophycocyanin; PE, R-phycoerythrin; Cy7, cyanine dye -7; GFP, green fluorescent protein; A647, Alexa Fluor 647.

Supplementary Table 2. Location of PC-3 metastasis per mouse. Legend: X within a box indicates the identification of lesions.

| Group | Mouse # | Femur | | Tibia | | Humerus | | Mandible | Spine | Pelvis | Lung | Liver |
| --- | --- | --- | --- | --- | --- | --- | --- | --- | --- | --- | --- | --- |
|  |  | Injected | Contralateral | Lateral | Contralateral | Lateral | Contralateral |  |  |  |  |  |
| **PC-3-DsRed** | 155 |  |  |  |  |  |  |  |  |  | **X** |  |
|  | 156 |  |  |  |  |  |  |  |  |  | **X** |  |
|  | 158 |  |  |  |  |  |  | **X** |  |  | **X** |  |
|  | 159 |  |  |  | **X** |  |  |  |  |  |  | **X** |
|  | 198 |  |  | **X** |  |  |  |  |  |  |  |  |
|  | 200 |  |  | **X** |  |  |  |  |  |  |  |  |
|  | 202 |  |  |  |  |  |  |  |  |  |  |  |
| **hBMSC-Luc/GFP**  **+ PC-3-DsRed** | 165 | **X** |  |  |  |  |  |  |  |  |  |  |
|  | 166 |  |  |  |  |  |  |  |  |  |  |  |
|  | 167 |  |  |  |  |  |  |  |  |  |  |  |
|  | 168 |  | **X** |  |  |  |  |  |  |  |  |  |
|  | 169 | **X** | **X** | **X** | **X** | **X** | **X** | **X** | **X** | **X** | **X** | **X** |
|  | 170 |  |  |  |  |  |  | **X** |  |  |  | **X** |
|  | 171 |  | **X** | **X** | **X** |  |  | **X** |  |  |  | **X** |
|  | 204 | **X** |  |  |  |  |  |  |  |  |  |  |
|  | 205 |  |  |  |  |  |  |  |  |  |  |  |
|  | 207 |  |  |  |  |  |  |  |  |  |  |  |
|  | 209 |  |  |  |  |  |  |  |  |  |  |  |
|  | 210 | **X** |  |  |  |  |  | **X** |  |  |  | **X** |
|  | 211 | **X** | **X** | **X** |  |  |  |  |  |  |  |  |

## Supplementary Figures

*
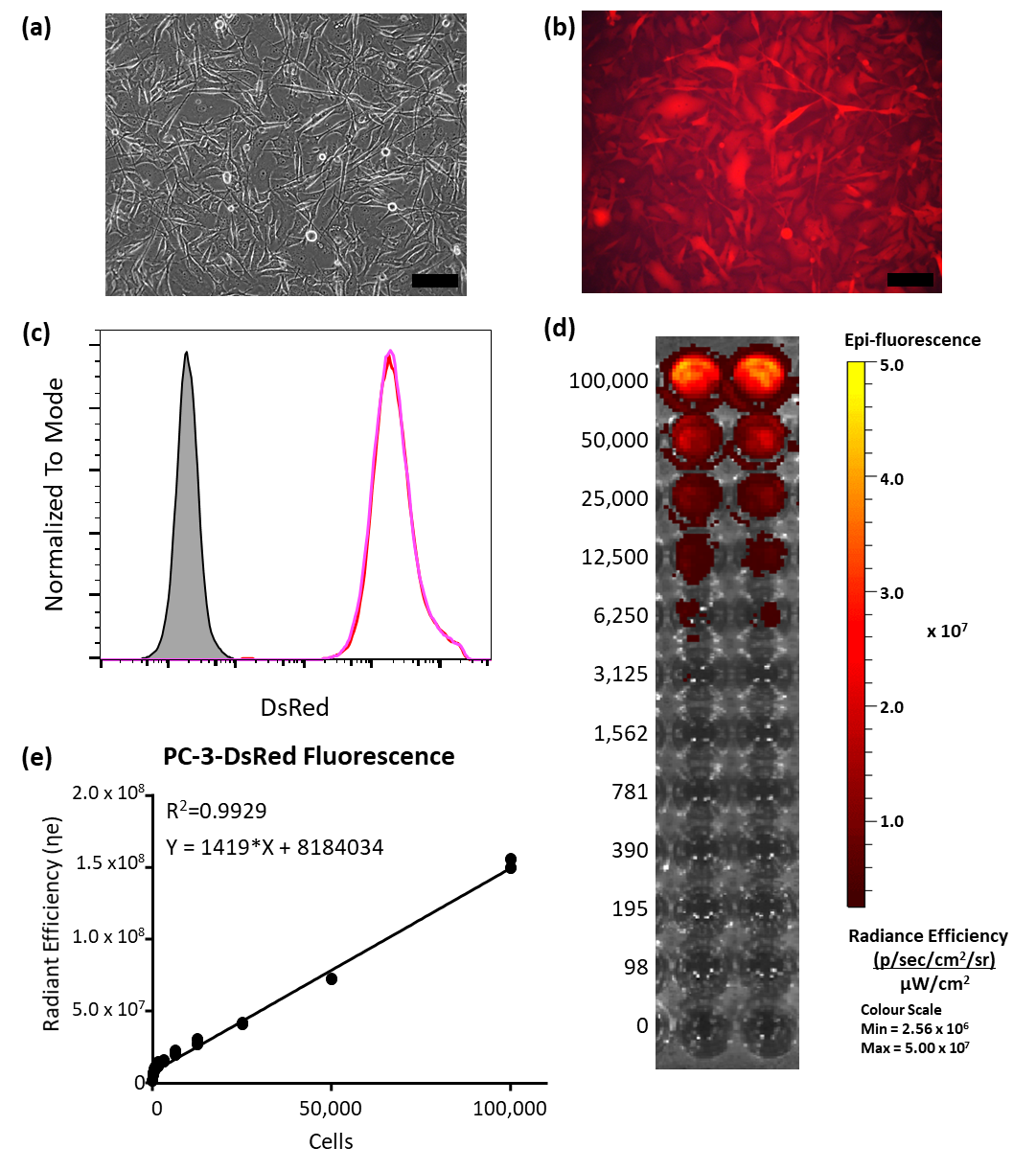
*

**Supplementary Figure 1.** PC-3-DsRed characterization. **(a)** Brightfield image of cells. **(b)** Fluorescence image of cells. Scale bar = 100 µm. **(c)** Flow cytometry analysis demonstrating similar fluorescence signal from PC-3-DsRed cells cultured with (pink) or without (red) antibiotic pressure, compared to non-transduced PC-3 cells (grey). **(d, e)** IVIS characterization of fluorescence signal from PC-3-DsRed titration in a 96 well plate. Statistics by Pearson correlation calculations demonstrating a linear relationship between cells seeded and fluorescence emission.


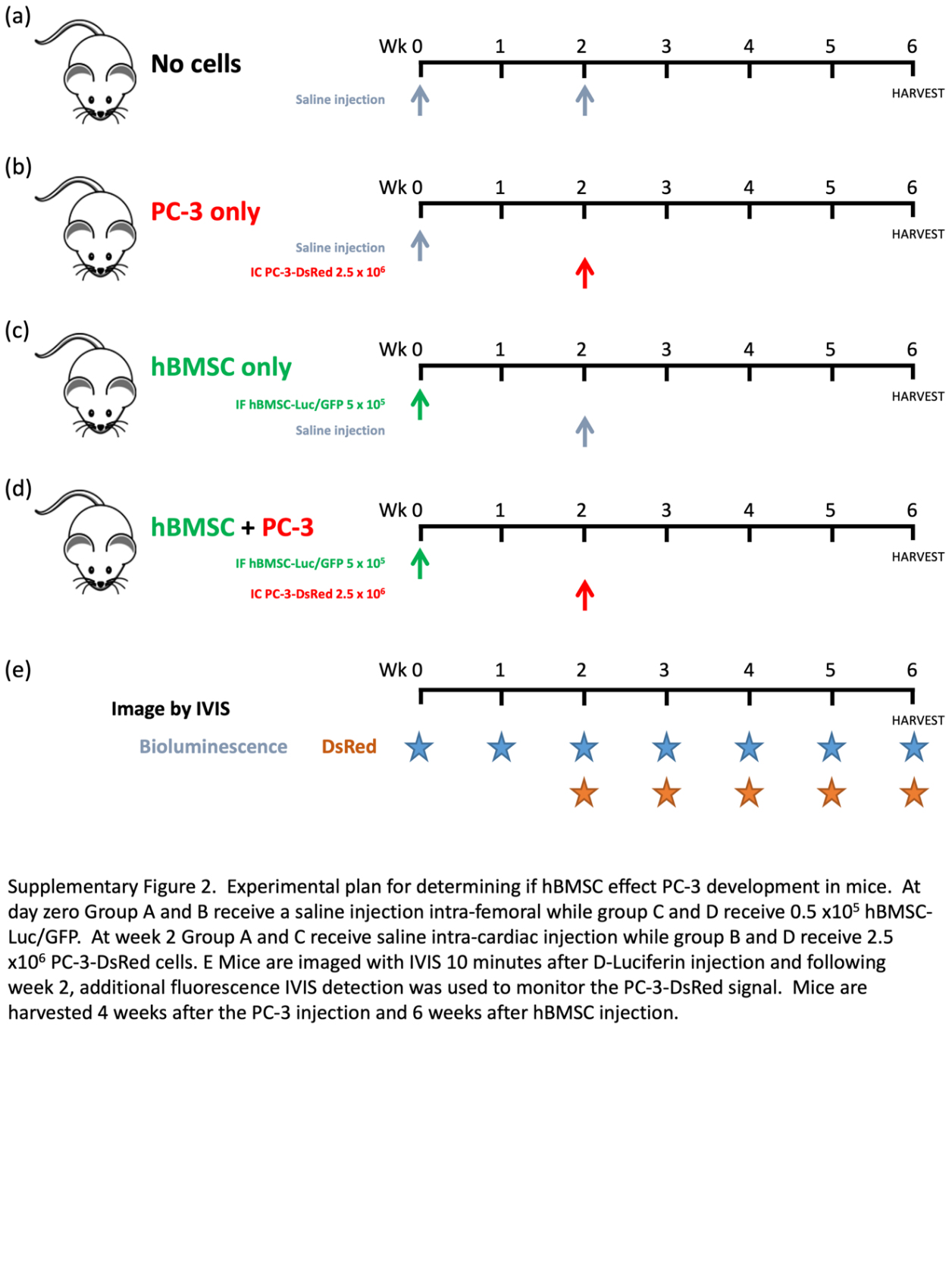


**Supplementary Figure 2.** Experimental timeline for characterization of hBMSC-Luc/GFP and PC-3-DsRed signal from mice. At day zero groups A and B received an intrafemoral media injection while groups C and D received 5 × 10^5^ hBMSC-Luc/GFP. At week 2 groups A and C received media intracardiac injection, while groups B and D received 2.5 × 10^6^ PC-3-DsRed. Mice were imaged with IVIS 10 minutes after D-Luciferin injection at the indicated time points. DsRed was imaged using the dual filters of background 500 nm vs DsRed filter at 570 nm. Mice were euthanised at week 6.


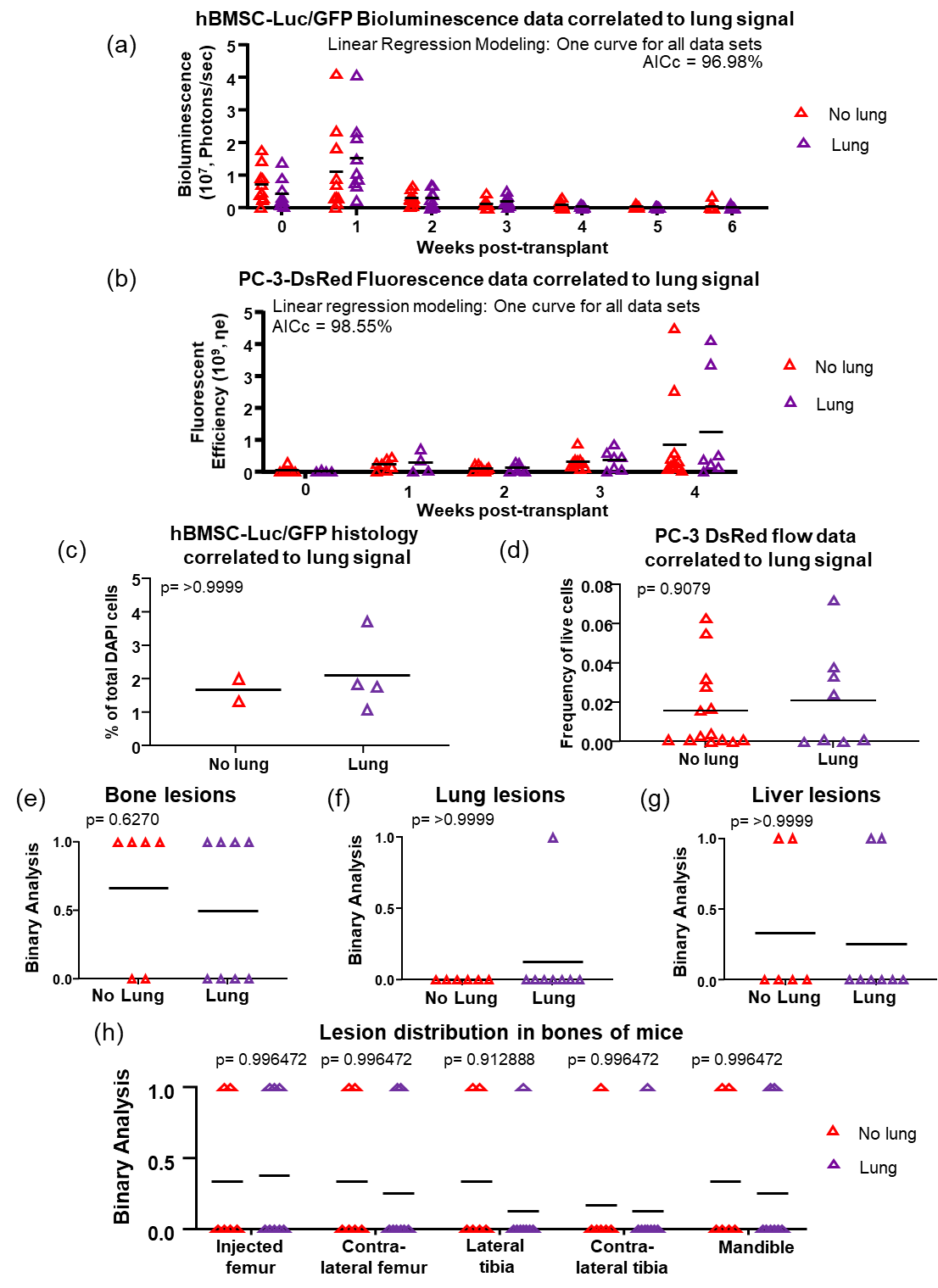


**Supplementary Figure 3.** Analysis of mice with (**Lung**) or without (**No Lung**) bioluminescence signal from the lung on the day of hBSMC-Luc/GFP transplant. **(a)** Bioluminescence signal from hBSMC-Luc/GFP in femurs over time. **(b)** PC-3-DsRed fluorescence over time, as detected by IVIS imaging. Linear regression utilized with AiCc determination of best fit. **(c)** hBMSC-Luc/GFP as detected by histology 6-weeks post-transplant and **(d)** flow data of PC-3-DsRed frequency over live cells. Binary analysis of lesion prevalence in mice within **(e)** bone, **(f)** lung, and **(g)** liver. Statistics completed with a Mann-Whitney test and p-value reported. **(h)** Bone lesion distribution in individual bones. Statistics: multiple t-tests with a Holm-Sidak's correction method with 0.05% threshold of significance. Outcomes were similar with or without bioluminescence signal from the lung on the day of hBSMC-Luc/GFP transplant.


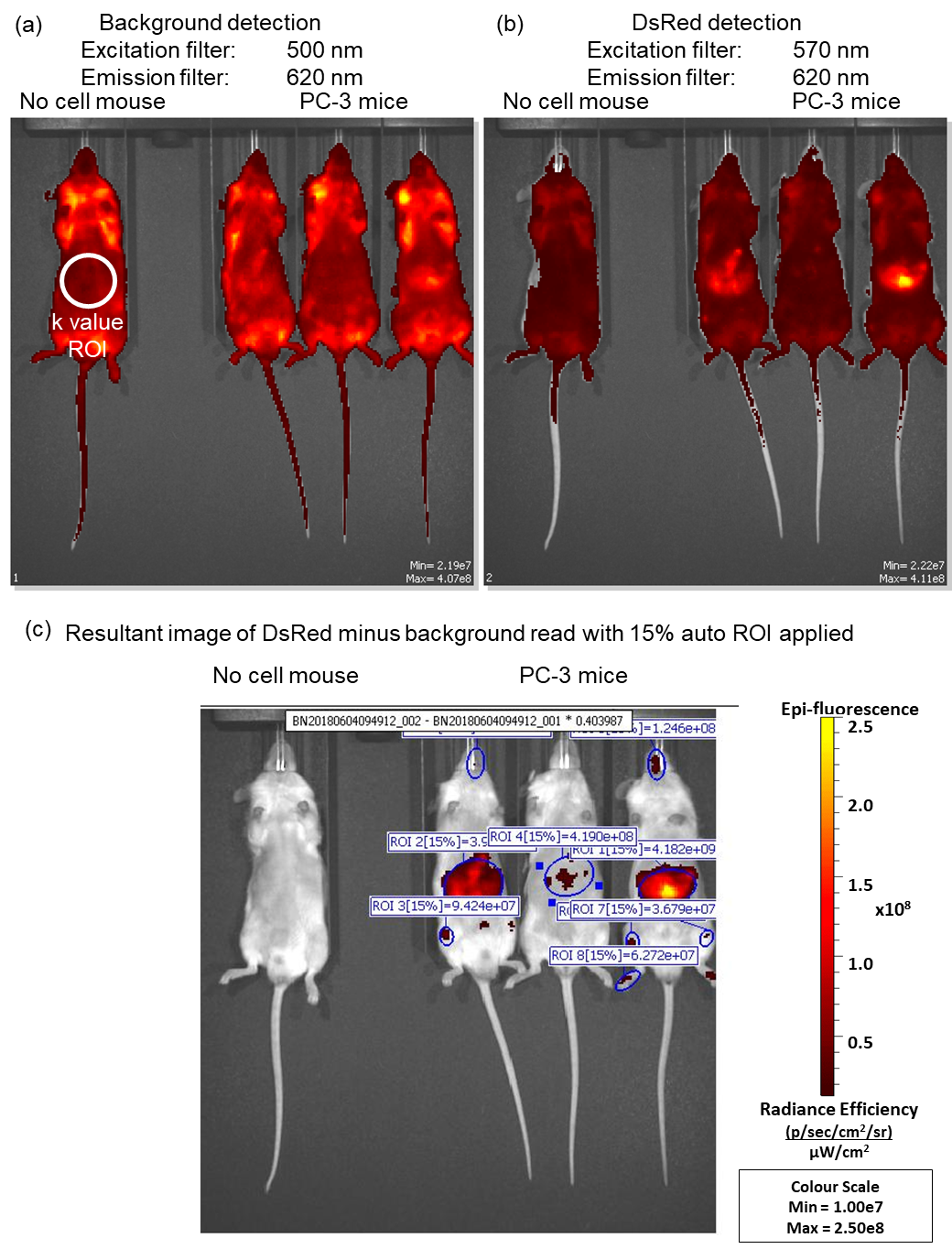


**Supplementary Figure 4**. DsRed fluorescence detection using the IVIS dual filter method. **(a)** Background image acquired using the 500 nm filter and the background ROI image from no cell mouse. **(b)** DsRed 570 nm image and **(c)** resultant image mathematics with auto threshold ROI resulting in quantification used in images.


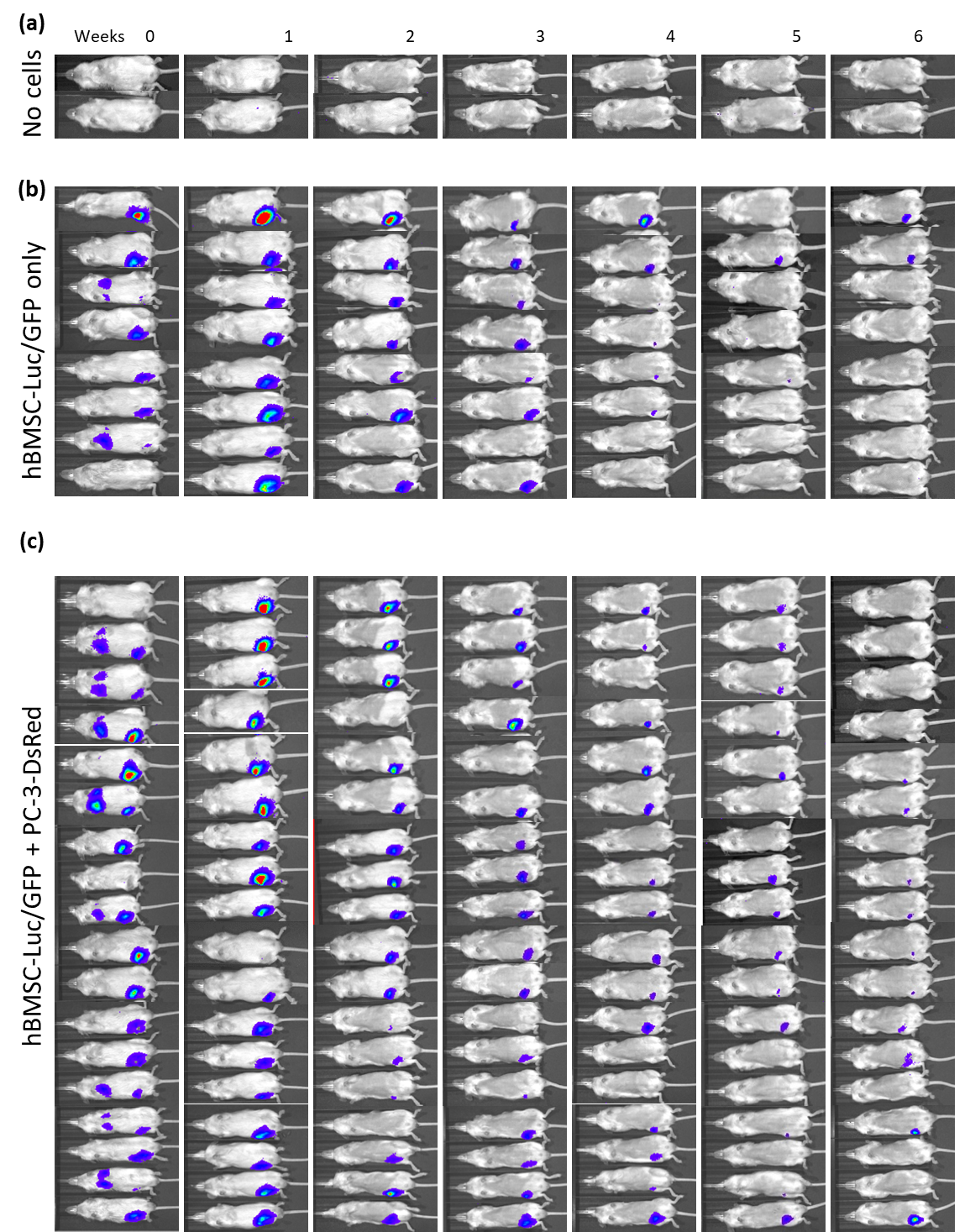


**Supplementary Figure 5.** Individual bioluminescence images of mice over time. **(a)** no cell group, n=2, **(b)** hBMSC-Luc/GFP only group n=8, **(c)** hBMSC-Luc/GFP + PC-3-DsRed n=18.


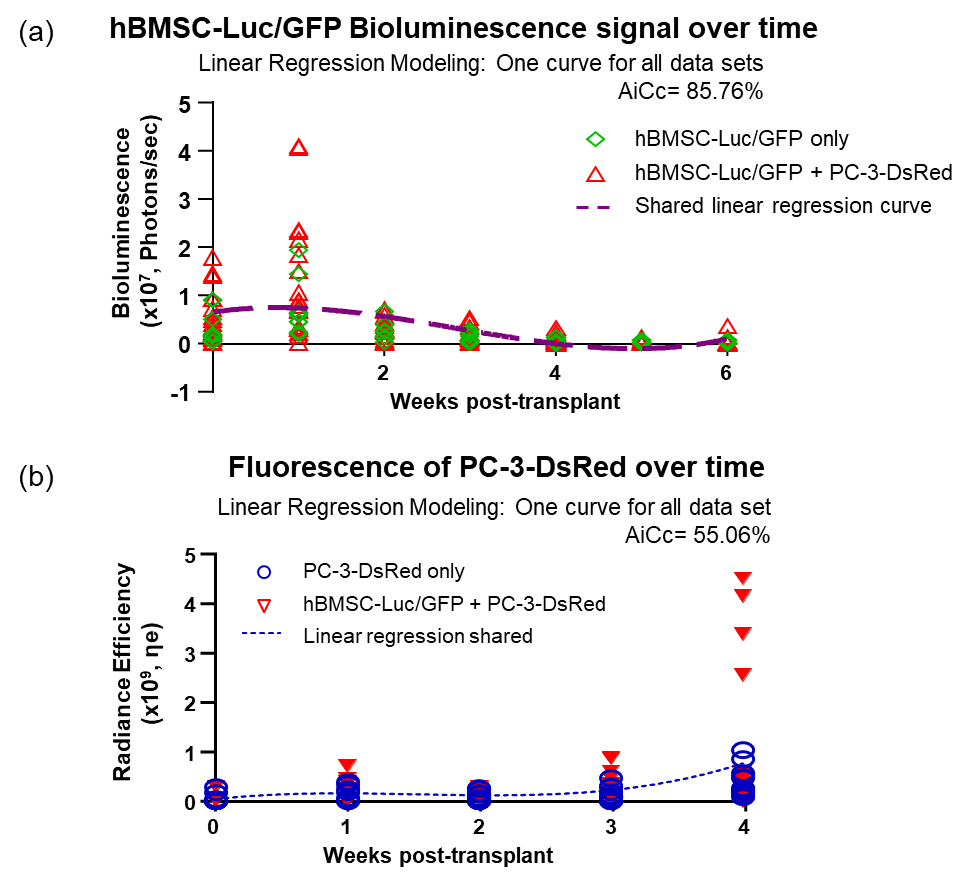


Supplementary Figure 6. Linear regression curves between groups. (a) hBMSC-Luc/GFP Bioluminescence signal over time. (8 mice with hBMSC-Luc/GFP (green), and 18 mice with hBMSC-Luc/GFP + PC-3-DsRed (red)). (b) PC-3-DsRed fluorescence over time. Linear regression performed and compared with AiCc fit, estimating the likelihood that a single curve fit both data sets (14 mice with PC-3-DsRed and 18 mice with hBMSC-Luc/GFP + PC-3-DsRed).


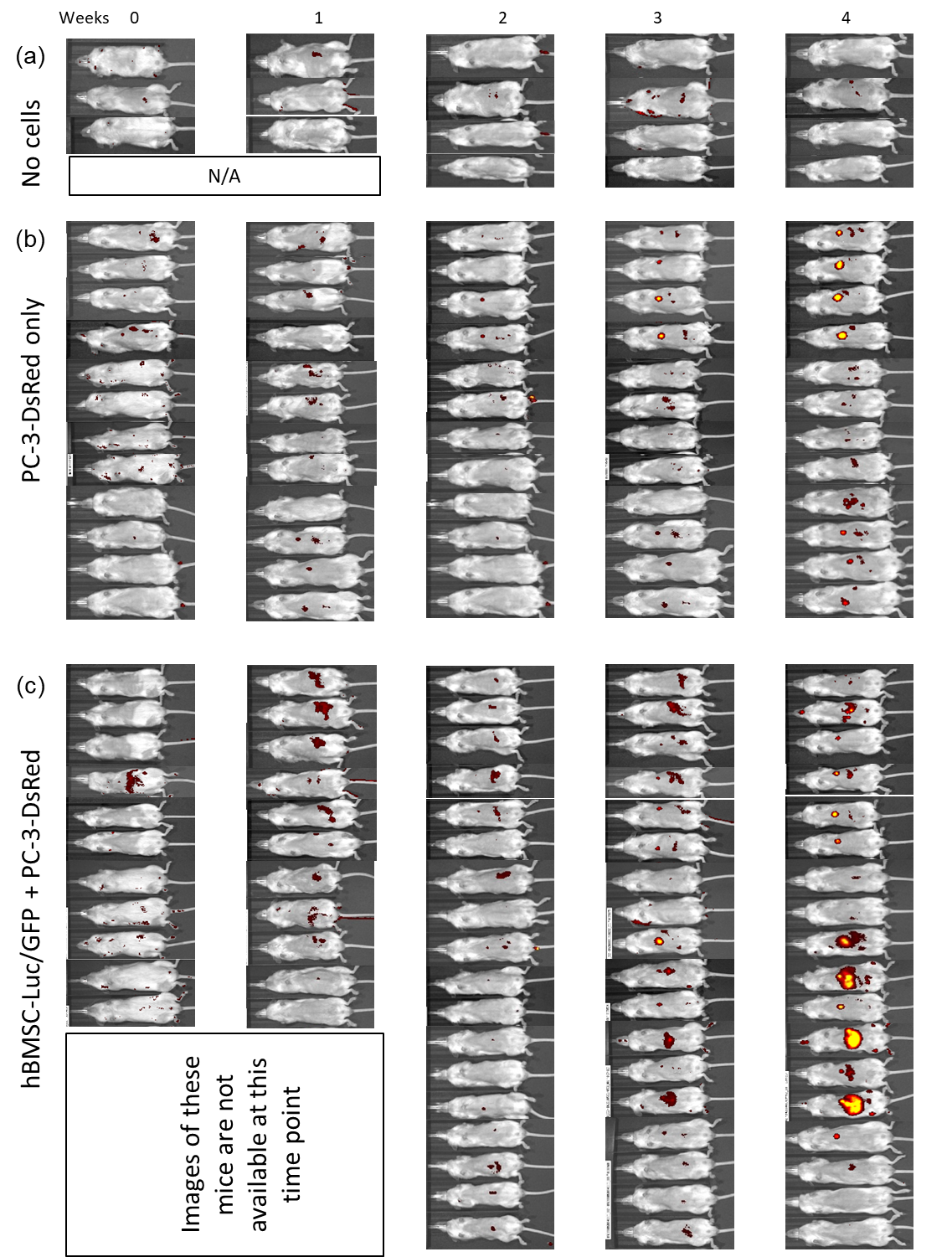


**Supplementary Figure 7.** Individual bioluminescence images of mice over time. **(a)** no cell group, n=4, **(b)** PC-3-DsRed only group n=12, **(c)** hBMSC-Luc/GFP + PC-3-DsRed n=18. Images of some mice were not captured due to technical issues.


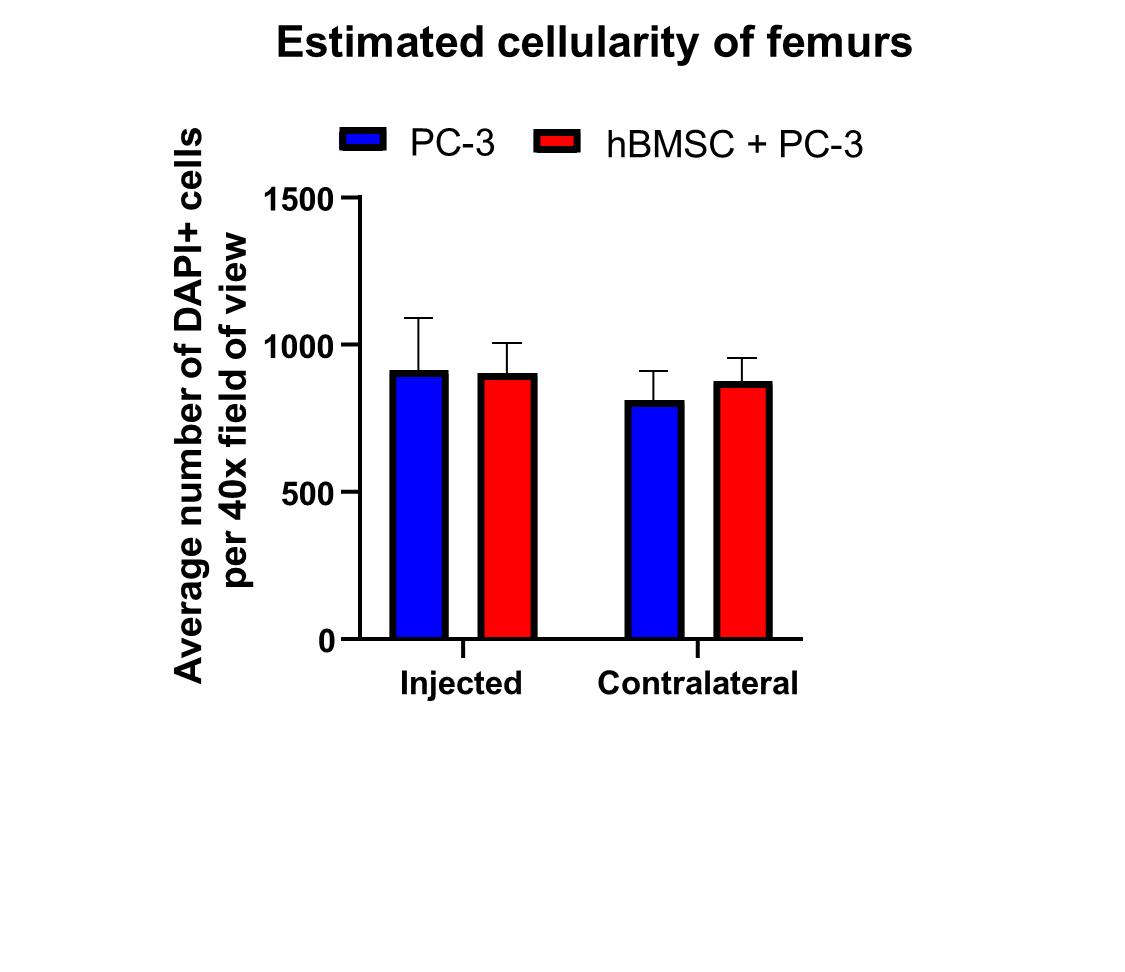


**Supplementary Figure 8.** Estimated cellularity of femur between groups with and without hBMSC and comparison of Injected vs Contralateral femur. Each mouse had 3 40X field of views collected and total cellularity estimated (DAPI^+^ cells). The average number was then used to generate the table above. Groups PC-3 n=4, hBMSC + PC-3 n=7. Statistic student t-test p=0.5898.


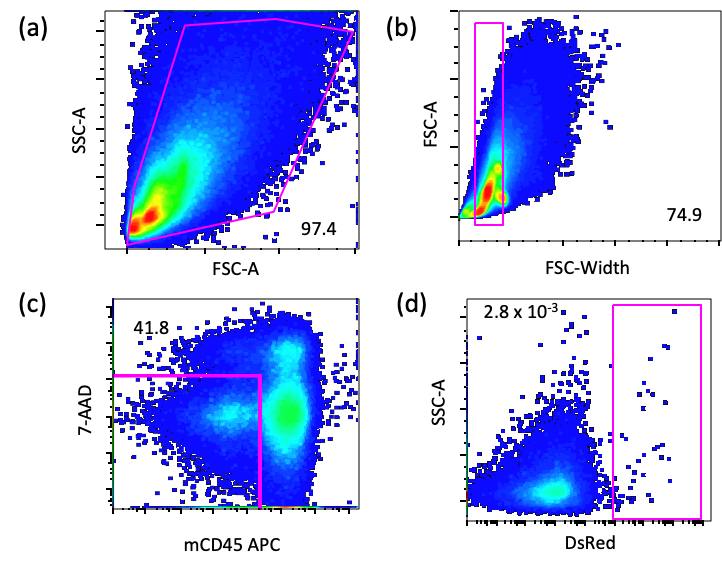


**Supplementary Figure 9.** Gating strategy for quantification of PC-3-DsRed frequency in femurs. (**a**) Forward and side scatter gating was taken to (**b**) Single-cell gate. (**c**) Live (7AAD^-^) and mouse CD45 negative cells were examined (**d**) for DsRed^+^ cell frequency. Results were analysed as a frequency of live mCD45^-^ cells.


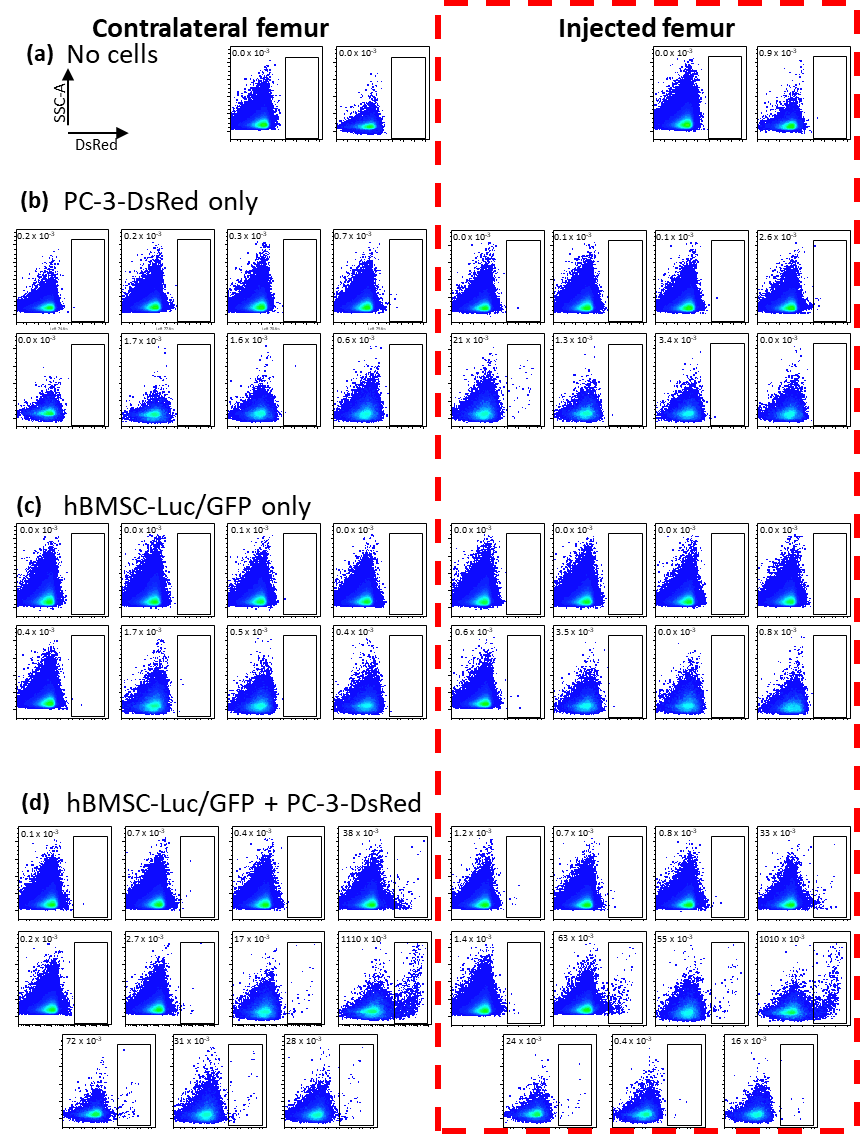


**Supplementary Figure 10.** Individual flow plots for femur analysis. Contralateral femur on left, Injected femur on the right in the red box. Cells were selected as in Figure S8 and presented as side-scatter vs DsRed. (**a**) No cell group, (**b**) PC-3-DsRed only, and (**c**) hBMSC-Luc/GFP only, and (**d**) combined hBMSC-Luc/GFP + PC-3-DsRed group. Data pooled from two experiments.


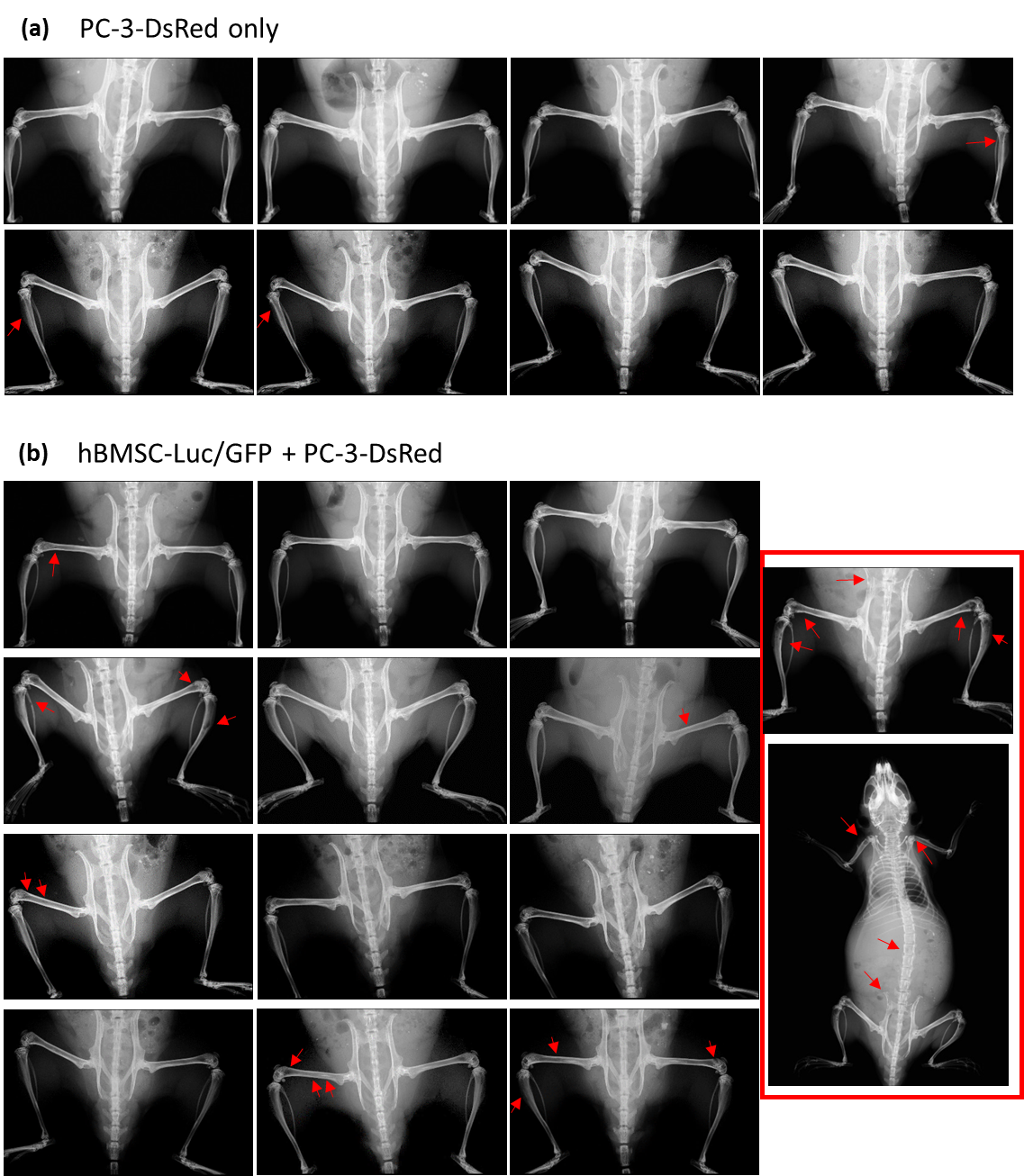


**Supplementary Figure 11.** X-ray of mice at harvest. A full X-ray image was taken of each mouse plus a focused image of the femurs. hBMSC-Luc/GFP were injected into the right femur of mice. (**a**) PC-3-DsRed only group, (**b**) hBMSC-Luc/GFP + PC-3-DsRed group. Red arrows indicated detected bone lesions in X-ray. Bone lesions were restricted to leg bones of all mice except one mouse (red box) who had additional lesions in the forelimb, vertebrae, and pelvis bones as indicated. PC-3-DsRed n=7, hBMSC-Luc/GFP + PC-3-DsRed n=13.


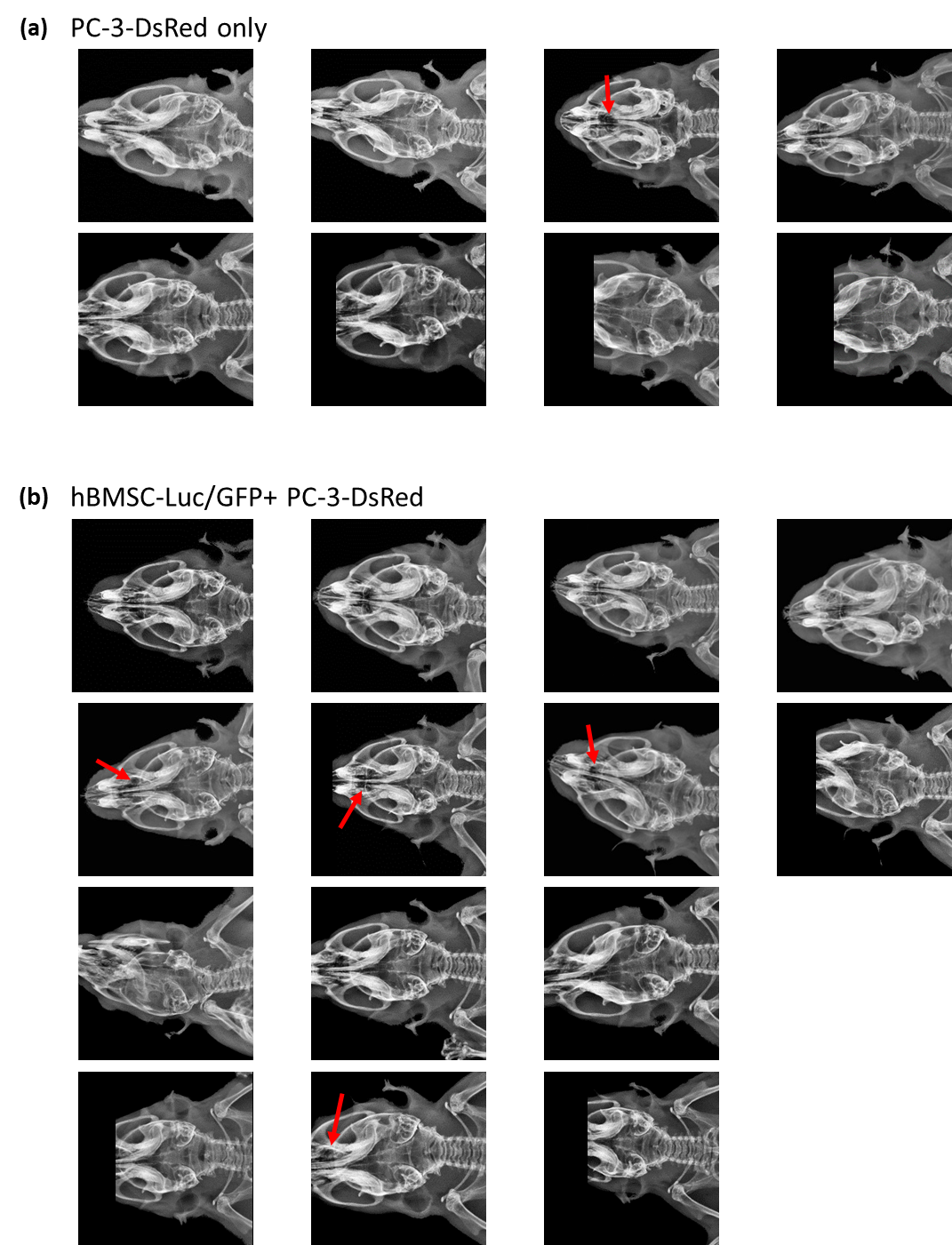


**Supplementary Figure 12.** X-ray of Mandibles in mice. **(a)** PC-3-DsRed group. **(b)** Animals that received hBMSC-Luc/GFP + PC-3-DsRed. Red arrows identified lesions. PC-3-DsRed n=7, hBMSC-Luc/GFP + PC-3-DsRed n=13.


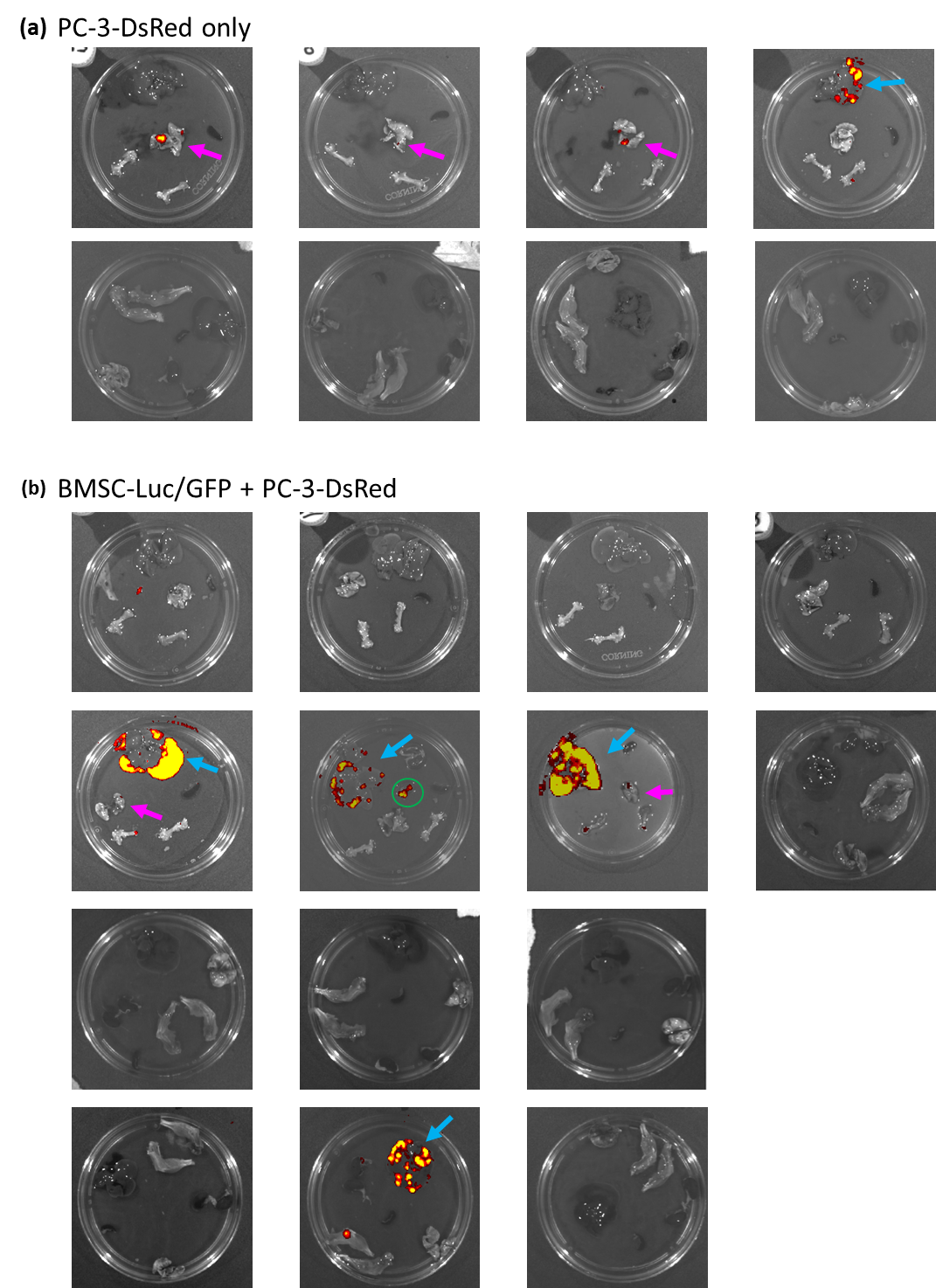


**Supplementary Figure 13.** DsRed signal in mouse organs. The spleen, femurs, liver, lung, and later renal glands were extracted from mice for imaging on the IVIS for DsRed using image maths. For Image Maths anlaysis background value was taken from the spleen. **(a)** Groups PC-3-DsRed only at the top, **(b)** hBMSC-Luc/GFP + PC-3-DsRed group in the bottom section. The purple arrows indicate lung lesions; blue arrows indicate liver lesions. Green circles highlight a heart that was tested. PC-3-DsRed n=7, hBMSC-Luc/GFP + PC-3-DsRed n=14.
